# Corneal changes in estrogen-dependent breast cancer after hormonal treatment

**DOI:** 10.1038/s41598-025-16065-7

**Published:** 2025-08-19

**Authors:** Mohamed Salah hamed Mohamed, Alaa Mahmoud, Haitham Thabit Abd Alkareem Rashdan, Elshimaa A.Mateen Mousa

**Affiliations:** Department of Ophthalmology, Faculty of Medicine, Sohag University, Sohag, Egypt

**Keywords:** Breast cancer, Estrogen, Hormonal treatment, Aromatase inhibitors, Corneal changes, Cancer, Breast cancer, Medical research, Outcomes research

## Abstract

Aromatase inhibitors (AI) therapy is the treatment of choice for estrogen receptor-positive breast cancer (BC). While AIs effectively suppress tumor growth, estrogen deprivation may have negative impacts on the eyes. This work evaluated the anterior segment changes in estrogen-dependent BC after hormonal treatment. This cross-sectional study was conducted on 40 female patients aged ≥ 18 who received treatment for BC. Patients were allocated to two groups: Group A served as the control group and did not receive AIs, while Group B received one of the AIs for BC treatment. Before treatment, there was no significant difference in tear break-up time (TBUT), Schirmer test (ST) score, and hexagonality. However, post-treatment TBUT and ST values were lower in Patients who received treatment. Corneal thickness, CCT, or back and front elevations were comparable before or after treatment. AI therapy in estrogen-dependent BC patients is associated with significant corneal changes, including potential corneal stress evidenced by impaired tear film function and reduced endothelial hexagonality, while corneal thickness and front and back elevation remain stable.

## Introduction

Breast cancer (BC) represents the most prevalent form of cancer among women^1^. Selective estrogen receptor modifiers (SERMs) such as tamoxifen, raloxifene, and toremifene, as well as aromatase inhibitors (AIs) like anastrozole, letrozole, and exemestane, are included in adjuvant hormonal therapy for BC in postmenopausal women^2^.

AIs inactivate aromatase, an enzyme that converts androgen to estrogen in peripheral tissues^3^. The development and progression of cancer cells are facilitated by estrogen through estrogen receptors (ER). As a result, the treatment and chemoprevention of BC are likely to be significantly influenced by the synthesis and action of estrogen^4^.

The influence of estrogen is facilitated via alpha and beta ER, both of which are present in the human retina^5^. Prior research has documented a variety of ocular adverse effects associated with AIs, including visual disturbances, retinal hemorrhages, hemiretinal artery occlusion, vitreoretinal traction, corneal epithelial lesions, and dry eye^6^.

All components located between the corneal epithelium and the posterior capsule of the lens are included in the anterior segment of the eye^7^.

Patients receiving treatment with AIs exhibit significant differences in visual acuity, retinal nerve fiber layer thickness, and visual evoked potentials compared to healthy individuals. These findings suggest that AIs may affect the retina and optic nerve function. This impact is likely due to the suppression of estrogen biosynthesis caused by AIs, which deprives the retina and choroid of estrogen’s protective benefits^8^.

There is a potential link between AIs and dry eye syndrome development. The conversion of androgens to estrogens may be impeded by AIs, which may contribute to ocular surface issues^9^. Also, a higher prevalence of dry eye syndrome is noted among women undergoing AI treatment, as demonstrated by increased ocular surface disease index (OSDI) scores^10^. Additionally, AI therapy has been associated with joint pain and sicca syndrome, both of which are believed to result from the significant estrogen deficiency induced by these drugs. Ocular surface diseases appear more frequently and with greater severity in patients undergoing AI treatment^11^.

To the best of our knowledge, there have been no prior investigations assessing the alterations in the anterior segment associated with estrogen-dependent BC following hormonal therapy. Thus, this work evaluated the anterior segment changes in estrogen-dependent BC after hormonal treatment.

## Patients and methods

This prospective cohort study was conducted on 40 female patients aged ≥ 18 who received hormonal therapy for BC. The research was conducted between june 2024 and january 2025, following the approval from Sohag University Hospital’s ethical committee (approval code: Soh-Med-24-03-14PD) and registration on ClinicalTrials.gov (ID NCT06924229) date: 11/4/2025). All methods were performed in accordance with the relevant guidelines and regulations. The patients provided informed written consent.

Candidates who had undergone ocular-surface surgery, ocular trauma, contact lens wear, diabetes mellitus, uncontrolled hypertension, topical-systemic medication, corneal toxicity, or any systemic disease that could negatively impact the corneal sub-basal nerve plexus were excluded from the study.

Patients were allocated into two groups: Group II contained women who had received one of the AI (anastrazol 1 mg once daily) for BC therapy, while Group I was made up of women who did not get AIs and acted as the control group.

All participants underwent a thorough evaluation that included demographic data collection, such as age, as well as a review of their past medical history. Data about tumor history, breast cancer treatment methods, duration of hormone therapy, and ocular symptoms—including blurred vision, redness, irritation or a foreign body sensation, excessive tearing, and sensitivity to light—were documented. Additionally, all patients underwent a thorough eye examination, which incorporated evaluations of visual acuity, intraocular pressure, and slit-lamp biomicroscopy.

Evaluations were conducted before the initiation of treatment and repeated at 3 and 6 months post-treatment. An observer also performed ocular surface tests for all participants.

A series of tests were carried out in sequential order in order to assess the state of the ocular surface. The Tear Break-Up Time (TBUT) test is a common procedure that involves the application of sterile fluorescein test strips that have been soaked in saline solution. Multiple blinks were performed by the patients, and the amount of time that passed between the last blink and the emergence of the first dry patch on the cornea was recorded as the time between blinks (TBUT). An evaluation of the health of the cornea and conjunctiva was performed using a staining technique called lissamine green (LG), with LG strips being put to the inferior conjunctival sac. Under the influence of white light, staining was evaluated, and scores were awarded between 0 and 5 in accordance with the Oxford Scheme^12^.

Tear production was assessed using the Schirmer-I test (ST), which was carried out under topical anesthetic (proparacaine hydrochloride for a concentration of 0.5%). While sterile filter paper strips were gently inserted into the lower fornix of the eyelid for a period of five minutes, patients were instructed to maintain a straight-ahead stare^13^. In addition, the OSDI questionnaire was used to evaluate the degree of severity of the subjective ocular symptoms that were experienced.

Further examinations included specular microscopy to evaluate endothelial changes by assessing central cell density, central corneal thickness, coefficient of variation, and hexagonality. Pentacam imaging was also performed using Scheimpflug imaging to analyze corneal changes through different maps, including sagittal curvature, corneal thickness, front elevation, and back elevation.

The study’s primary outcome was hexagonality, while secondary outcomes included foreign body sensation, blurred vision, tearing, redness, and photophobia.

### Statistical analysis

The statistical analysis was performed using SPSS version 26 (IBM Inc., Chicago, IL, USA). The normality of data distribution was determined using the Shapiro–Wilk test and histogram analysis. An unpaired Student’s t-test was used to compare parametric quantitative variables (mean ± SD) between two groups. Non-parametric quantitative data were presented as median and interquartile range (IQR) and examined using the Mann–Whitney test. Qualitative variables were provided as frequencies and percentages (%) and analyzed using the Chi-square test or Fisher’s exact test, if applicable. A two-tailed p-value < 0.05 was deemed statistically significant.

## Results

**Fig. 1:**
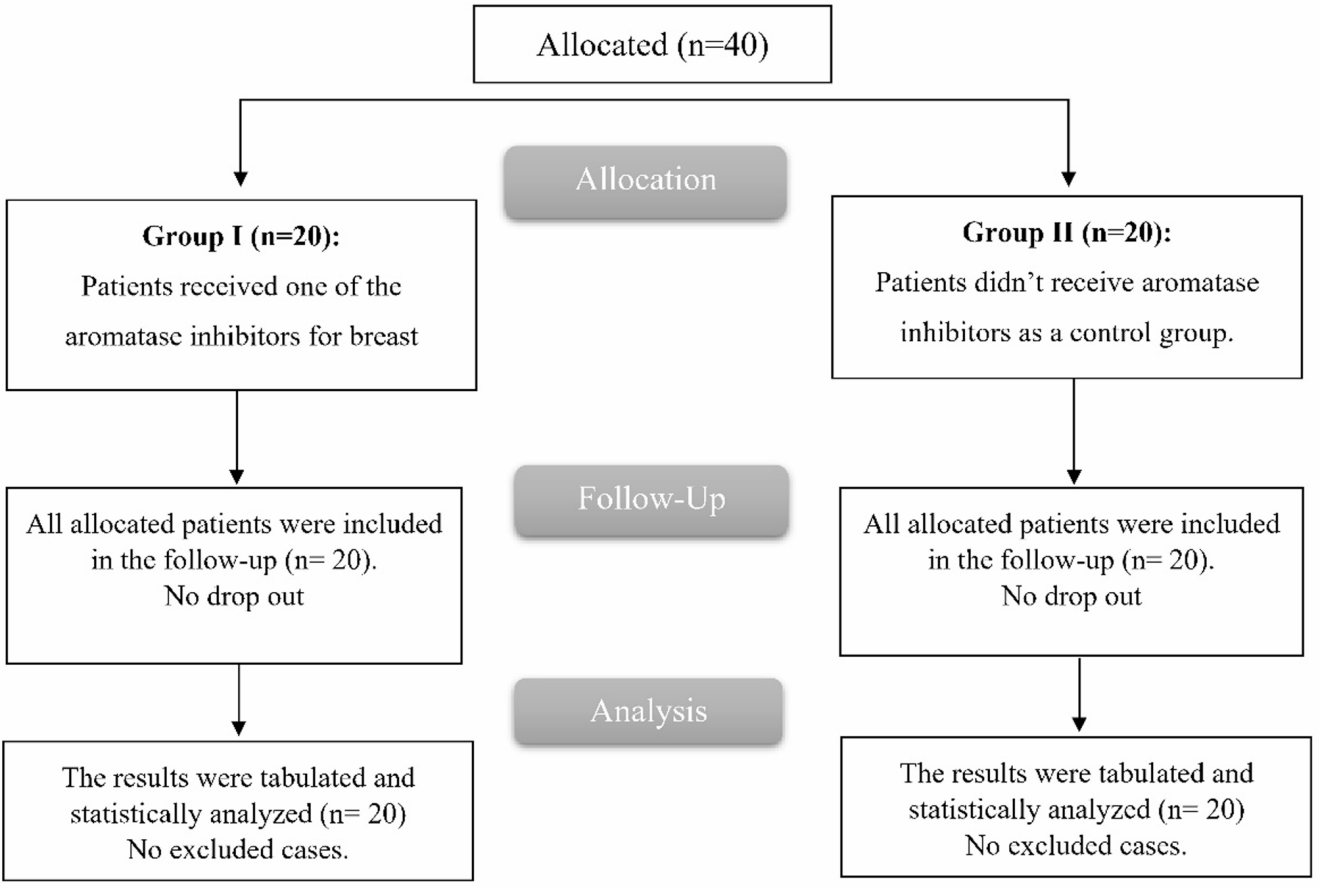
STROBE flowchart of the enrolled patients.

As part of this study, forty patients were split into two equal groups, each consisting of twenty individuals. All of the patients who were allocated to the study were observed and statistically evaluated.Data of post treatment at 3 and 6 months were combined single post treatment dataset due to insignificant difference Fig. 1.

Demographics were comparable between the two groups (Table 1).

**Table 1:** Demographics of the studied groups.

		Group I (*n* = 20)	Group II (*n* = 20)	*P*
Age (years)		31.55 ± 5.76	30.4 ± 5.55	0.524
Medical history	Diabetes mellitus	2(10%)	5(25%)	0.407
	Hypertension	8(40%)	6(30%)	0.740
	Hypercholesterolemia	7(35%)	5(25%)	0.730
	Hyperthyroidism	3(15%)	4(20%)	0.677
Breast cancer treatment modalities	Surgery	3(15%)	4(20%)	0.677
	Chemotherapy	6(30%)	7(35%)	0.735
	Radiotherapy	2(10%)	3(15%)	1
	Aromatase inhibitors	9(45%)	6(30%)	0.513
Duration of hormonal treatment		20(10–28)	22(12–30)	0.481

Data is presented as mean ± SD or median (IQR).

There was no significant difference in pretreatment for TBUT and Schirmer tests across groups. However, group I had considerably lower levels than group II (*P* = 0.022 and 0.016 respectively) (Table 2).

**Table 2:** TBUT and schirmer test of the studied groups.

	Group I (*n* = 20)	Group II (*n* = 20)	*P*
TBUT			
Pre-treatment	8.65 ± 2.06	9.65 ± 1.57	0.092
Post-treatment	9.75 ± 1.45	10.8 ± 1.32	0.022*
Schirmer test			
Pre-treatment	8.2 ± 2.17	9.05 ± 1.39	0.148
Post-treatment	10.15 ± 1.53	11.25 ± 1.21	0.016*

Data is presented as mean ± SD. TBUT: Tear break up time test.

Corneal thickness or CCT were similar between the two groups before and after therapy. Hexagonality was similar between the two groups before treatment, although it was significantly lower in group I than in group II (*P* = 0.038) (Table 3).

**Table 3:** Corneal thickness, CCT and hexagonality of the studied groups.

	Group I (*n* = 20)	Group II (*n* = 20)	*P*
Corneal thickness (µm)			
Pre-treatment	493.25 ± 24.11	484.75 ± 39.75	0.419
Post-treatment	491.4 ± 22.69	497.15 ± 15.28	0.353
CCT (µm)			
Pre-treatment	2029.5 ± 123.48	2068.3 ± 68.43	0.227
Post-treatment	2065.15 ± 116.13	2074.8 ± 69.35	0.751
Hexagonality			
Pre-treatment	54.05 ± 7.32	57.3 ± 2.89	0.073
Post-treatment	55.45 ± 4.84	58.15 ± 2.85	0.038*

Data is presented as mean ± SD. *Significant when *P* ≤ 0.05. *CCT* Central corneal thickness.

The back and front elevations were comparable before and after treatment in both groups (Table 4).

**Table 4:** Back and front elevation of the studied groups.

	Group I (*n* = 20)	Group II (*n* = 20)	*P*
Back elevation			
Pre-treatment	13.6 ± 4.85	11.65 ± 2.8	0.128
Post-treatment	12.15 ± 2.92	10.95 ± 2.54	0.174
Front elevation			
Pre-treatment	14.85 ± 3.07	12.75 ± 3.92	0.067
Post-treatment	12.95 ± 3.44	11.25 ± 2.83	0.096

Data is presented as mean ± SD.

## Discussion

The retina and the choroid, in addition to the lacrimal glands, which include the Meibomian glands, the conjunctival and corneal surfaces, have been shown to contain both alpha and beta ER cells^14^.

In order to maintain a normal lacrimal film and ensure that the ocular surface is healthy, it is necessary to maintain a delicate balance between androgens and estrogens. Estrogens have a trophic function at the cornea, impact the aqueous component of the lacrimal layer, and stimulate the maturation of the superficial cells in the conjunctiva. Estrogens also have a role in the development of skin cells. Conversely, estrogens may induce inflammation at the ocular surface.

17beta estradiol increases mRNA levels of IL-1β, IL-6, IL-8, and GM-CSF in the cornea, potentially contributing to inflammation in dry eye illness^15^.

Androgens reduce inflammation and promote the synthesis of meibomian glands, which adds to the lacrimal film’s lipid layer. This layer contributes to stability and reduces evaporation, therefore preserving the ocular surface^16^.

BC patients treated with AIs had significantly lower peripheral estrogen levels, depriving the ocular surface of estrogen’s trophic protective actions. Although most occurrences of dry eye and Meibomian gland dysfunction are modest to moderate, an active screening by OSDI may enhance patients’ quality of life by highlighting the need for therapy to lessen visual impairment^17^.

We noted no significant difference in TBUT, ST scores, and hexagonality before treatment. However, post-treatment, TBUT, and ST scores were lower in the control group compared with the AI group. There was also no significant difference in corneal thickness, CCT, or back and front elevations before or after treatment.

AIs function by inhibiting the aromatase enzyme, leading to a significant reduction in estrogen levels. This decrease can impair the function of the lacrimal and meibomian glands, resulting in reduced tear secretion and altered tear film composition, thereby contributing to dry eye symptoms^18^.

AIs are designed to lower systemic estrogen levels to prevent the growth of estrogen-dependent BC cells. However, this systemic depletion also affects estrogen-dependent processes in other tissues, including those maintaining ocular surface integrity. The reduced estrogen levels can lead to decreased goblet cell density and mucin production, further destabilizing the tear film^18^.

Estrogen has been shown to exert protective effects on various cell types, including corneal endothelial cells. The hormone promotes cell survival and function through antioxidant properties and modulation of apoptotic pathways. The estrogen depletion caused by AIs may lead to increased oxidative stress and apoptosis in corneal endothelial cells, resulting in altered cell morphology and decreased hexagonality^18^.

Estrogen is known to influence vascular function, including ocular blood flow. Reduced estrogen levels may impair ocular perfusion, leading to hypoxic conditions that can adversely affect corneal endothelial cell health and morphology^18^.

In accordance with our findings, Ağın and colleagues^19^, reported that the densities of the long and total sub-basal nerve densities, as well as the TBUT, ST scores, basal epithelium, anterior and posterior keratocytes, and endothelial cell densities, were found to decrease, while the meiboscore and corneal sensitivity were found to increase during the treatment. At both the three-month and six-month timepoints, the rates of endothelial pleomorphism were significantly lower than they were before the therapy.

Also, Bicer et al.^11^ demonstrated that the control group had considerably higher post-treatment TBUT and ST scores than the AI group did. This was the case throughout the study.

Additionally, Gibson et al.^17^ found that raised ocular symptoms were reported in both the AI-treated and untreated groups, and there was no discernible difference between the two groups. The meibum expressibility scores of women who were getting AI therapy for early-stage breast cancer were lower, and their pain perception was higher, as compared to a group of women who were not receiving treatment.

Moreover, Chatziralli et al.^20^ found that patients who were receiving AIs had a significantly higher incidence of ocular surface disease-related symptoms and signs as compared to healthy controls.

This study has several limitations. The small sample size and short follow-up duration (6 months) may limit the results’ generalizability and prevent long-term corneal change detection. The absence of baseline pre-treatment ocular surface data complicates causal attribution to AI therapy. Serum and ocular estrogen levels were not measured, preventing a direct correlation between hormonal status and ocular changes. The exclusion of patients with comorbidities like diabetes may underrepresent the actual clinical burden in broader populations.

## Conclusions

AIs therapy in estrogen-dependent BC patients is associated with significant corneal changes, including potential corneal stress evidenced by impaired tear film stability and affection of endothelial hexagonality. In contrast, corneal thickness and front and back elevation remain stable.

## Data Availability

The data that support findings of this study are available from third party (Sohag University) but restrictions apply to the availability of these data, which were used under license for the current study, and so arenot publicly available. Data are however available from the authors upon reasonable request and with permission of the third party. The corresponding author can be contacted if someone wants to request the data of this study.
